# Structure of a heterogeneous, glycosylated, lipid-bound, *in vivo*-grown protein crystal at atomic resolution from the viviparous cockroach *Diploptera punctata*

**DOI:** 10.1107/S2052252516008903

**Published:** 2016-06-27

**Authors:** Sanchari Banerjee, Nathan P. Coussens, François-Xavier Gallat, Nitish Sathyanarayanan, Jandhyam Srikanth, Koichiro J. Yagi, James S. S. Gray, Stephen S. Tobe, Barbara Stay, Leonard M. G. Chavas, Subramanian Ramaswamy

**Affiliations:** a Institute of Stem Cell Biology and Regenerative Medicine, Bellary Road, GKVK Campus, Bangalore, Karnataka 560 065, India; b Department of Biochemistry, Carver College of Medicine, University of Iowa, Iowa City, IA 52242, USA; c National Center for Advancing Translational Sciences, National Institutes of Health, 9800 Medical Center Drive, Rockville, MD 20850, USA; d Structural Biology Research Centre, High Energy Accelerator Research Organization, Tsukuba, Ibaraki 305-0801, Japan; e Centre for Cellular and Molecular Platforms, Bellary Road, GKVK Campus, Bangalore, Karnataka 560 065, India; f Department of Cell and Systems Biology, University of Toronto, Toronto, ON M5S 3G5, Canada; g Bio-Research Products Inc., Cherry Street, North Liberty, IA 52317, USA; h Department of Biology, University of Iowa, Iowa City, IA 52242, USA; i Experimental Division, Synchrotron SOLEIL, BP 48, L’Orme des Merisiers, 91192 Gif-sur-Yvette, France

**Keywords:** sulfur-SAD, glycosylation, viviparity in cockroach, protein heterogeneity

## Abstract

Macromolecular crystals for X-ray diffraction studies are typically grown *in vitro* from pure and homogeneous samples; however, there are examples of protein crystals that have been identified *in vivo*. Recent developments in micro-crystallography techniques and the advent of X-ray free-electron lasers have allowed the determination of several protein structures from crystals grown *in cellulo*. Here, an atomic resolution (1.2 Å) crystal structure is reported of heterogeneous milk proteins grown inside a living organism in their functional niche. These *in vivo*-grown crystals were isolated from the midgut of an embryo within the only known viviparous cockroach, *Diploptera punctata*. The milk proteins crystallized in space group *P*1, and a structure was determined by anomalous dispersion from the native S atoms. The data revealed glycosylated proteins that adopt a lipocalin fold, bind lipids and organize to form a tightly packed crystalline lattice. A single crystal is estimated to contain more than three times the energy of an equivalent mass of dairy milk. This unique storage form of nourishment for developing embryos allows access to a constant supply of complete nutrients. Notably, the crystalline cockroach-milk proteins are highly heterogeneous with respect to amino-acid sequence, glycosylation and bound fatty-acid composition. These data present a unique example of protein heterogeneity within a single *in vivo*-grown crystal of a natural protein in its native environment at atomic resolution.

## Abstract 2

This article presents the features and structures of protein crystals naturally grown *in vivo* within developing embryos of the only known viviparous cockroach, *D. punctata*. This study reveals the heterogeneous nature of the crystalline protein with respect to amino-acid sequence, glycosylation and bound fatty acid at atomic resolution.

## 1. Introduction   

Viviparity, the maternal nourishment of embryos during development, is a highly evolved type of reproduction that occurs in many groups of animals. Cockroaches have evolved over the past 320 million years (Garwood & Sutton, 2010 ▸; Garwood *et al.*, 2012 ▸). An interesting feature of their evolution lies in their mode of reproduction. There are three general types of cockroaches: oviparous, ovoviviparous and viviparous (Roth, 1970 ▸). The oviparous species (*e.g. Periplaneta ameri­cana*) either deposit the ootheca (enclosing the fertilized eggs) onto a substrate or retain them, extruded and attached to the female’s body (Roth & Willis, 1954 ▸). The ovoviviparous species (*e.g. Rhyparobia maderae*) deposit the ootheca in the brood sac of the female. In this brood sac or uterus, the embryos are provided with protection and water, but not with nutrients (Nalepa & Bell, 1997 ▸). *Diploptera punctata* is the only known viviparous cockroach, an evolutionarily advanced condition in which the eggs have little yolk, but the developing offspring are nourished directly by the mother from the brood sac wall. Viviparity enhances larval development, because the time to reproductive maturity is substantially reduced in *D. punctata* relative to ovoviviparous species (Roth & Willis, 1954 ▸; Willis *et al.*, 1958 ▸; Stay & Coop, 1973 ▸, 1974 ▸; Roth, 1989 ▸). Utilizing the sparse yolk, *D. punctata* embryos quickly develop strong pharyngeal muscles and a simple gut, enabling them to imbibe and deposit in their midguts a protein-rich liquid milk secreted by the brood sac (Stay & Coop, 1973 ▸, 1974 ▸; Evans & Stay, 1989 ▸). This milk provides a 60-fold whole-body increase in protein during embryonic development (Stay & Coop, 1973 ▸). Complementary DNA analyses revealed 22 distinct but similar peptides encoded by milk genes with homology to the lipocalin family of lipid-binding proteins (Williford *et al.*, 2004 ▸), which are referred to as lipocalin-like milk proteins or Lili-Mip in this article. Soon after ingestion of the liquid milk, protein crystals develop within the embryo midgut (Ingram *et al.*, 1977 ▸). The crystals were shown to contain milk glycoproteins, although less glycosylated than at the time of secretion from the brood sac (Ingram *et al.*, 1977 ▸; Williford *et al.*, 2004 ▸). Thus, viviparity in *D. punctata* involves the evolution of a milk-secreting brood sac and rapid development of embryos that are able to drink and, importantly, store complete nutrients (protein, carbohydrate and lipid) concentrated in crystalline form. The properties of these *in vivo*-grown milk protein crystals are associated with the evolution of viviparity in cockroaches and are the subject of the current study.

*In vivo*-grown protein crystals have been identified from a diverse group of organisms (Doye & Poon, 2006 ▸; Lange *et al.*, 1982 ▸; Dogan *et al.*, 2012 ▸; Pande *et al.*, 2001 ▸). Their presence inside cells has been linked to biological functions such as insulin secretion (Dodson & Steiner, 1998 ▸), sorting of secretory proteins in the Golgi apparatus (Arvan & Castle, 1998 ▸), pathogenicity in *Bacillus thuringiensis* (van Frankenhuyzen, 2013 ▸), storage mechanisms for infectious viruses (Coulibaly *et al.*, 2005 ▸, 2007 ▸, 2009 ▸) and for developmental proteins in seeds (Doye & Poon, 2006 ▸) and eggs (Papassideri *et al.*, 2007 ▸; Snigirevskaya *et al.*, 1997 ▸; Lange *et al.*, 1982 ▸). In humans, naturally occurring crystals have been associated with disease conditions including histiocytosis (Dogan *et al.*, 2012 ▸), hemoglobin C (Doye & Poon, 2006 ▸) and cataracts (Pande *et al.*, 2001 ▸). In these conditions crystal growth might be coincidental, but is associated with pathology. In this report, our analysis of Lili-Mip crystals shows that they contain a heterogeneous mixture of amino-acid sequences *in vivo* and diffract to atomic resolution.

Macromolecular crystals for X-ray diffraction studies are typically grown from pure and homogeneous samples. Heterogeneity from post-translational modifications is considered to significantly reduce the probability of obtaining well diffracting crystals. In the case of glycosylation, which is heterogeneous by nature, great efforts are made to deglycosylate proteins of interest to favour chemically homogeneous and structurally monodisperse molecules prior to crystallization. Anecdotally, chemists and early biochemists used crystallization to isolate single-molecular species.

The number of X-ray crystal structures that have been determined from *in vivo*-grown crystals is low. The major challenge in their structure determination lies in the handling of such crystals at third-generation X-ray sources owing to their small physical dimensions (Koopmann *et al.*, 2012 ▸). Crystal structures of baculovirus polyhedra have been determined up to 2.2 Å resolution from microcrystals grown *in vivo* (Coulibaly *et al.*, 2009 ▸). Baculovirus expression systems have been utilized to induce intracellular crystallization of cathepsin B from *Trypanosoma brucei* (TbCatB) and *Cytoplasmic polyhedrosis virus* (CPV) polyhedra from *Bombyx mori*, thereby allowing structure determination of TbCatB to 2.1 Å resolution (Redecke *et al.*, 2013 ▸; Koopmann *et al.*, 2012 ▸) and of CPV to 2.0 Å resolution (Coulibaly *et al.*, 2007 ▸). *In vivo*-grown crystals have also recently been interrogated by serial femtosecond crystallography (SFX) at X-ray free-electron laser (XFEL) sources as a potential solution for solving structures of systems that are not amenable to conventional crystallo­graphy, such as macromolecular complexes and chemically untreated proteins (Gallat *et al.*, 2014 ▸). The structure of *Bacillus thuringiensis* Cry3A toxin from *in vivo*-grown crystals has been determined directly from the bacterial cells using SFX (Sawaya *et al.*, 2014 ▸). With the exception of CPV, none of these proteins crystallized within their functional niche.

**Figure 1:**
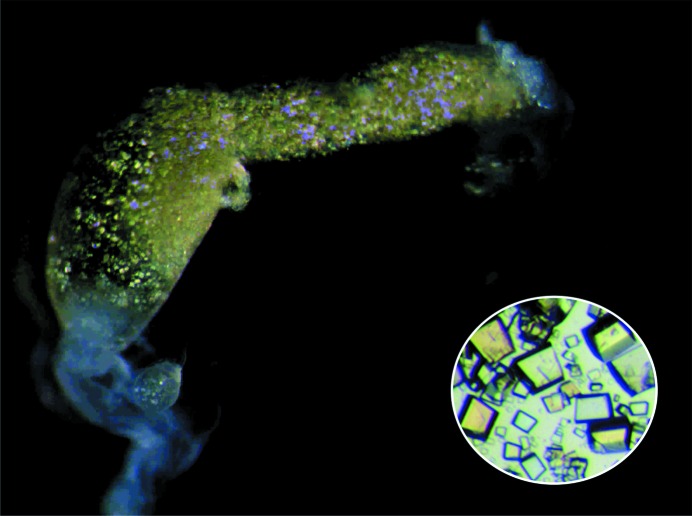
*In vivo*-grown Lili-Mip crystals from *D. punctata*. Polarized microscopy reveals birefringent protein crystals enclosed inside the embryo midgut and an enlarged view of the extracted crystals (inset).

All of the crystals described above could be called *in cellulo* crystals. In comparison to *in cellulo*-grown crystals, relatively large protein crystals (up to 10 × 10 × 30 µm) were identified in the midgut (*in vivo*) of developing embryos of the cockroach *D. punctata* (Fig. 1 ▸; Ingram *et al.*, 1977 ▸). While the cytoplasmic volumes of cells impose size constraints on protein crystals grown *in cellulo*, the substantially larger volume of the cockroach midgut allows larger crystals to develop. Surprisingly, these protein crystals diffracted to 1.2 Å resolution and we report the first structure of a naturally occurring and chemically unaltered, heterogeneous protein crystal grown *in vivo* at atomic resolution.

## 2. Materials and methods   

### 2.1. Crystal isolation from *in vivo* conditions   

Crystals were extracted from *D. punctata* embryo midguts. The cockroaches, which were fed Lab Chow (Purina, St Louis, Missouri, USA) and water, were maintained at an ambient temperature of 27°C, with a light and dark cycle of 12 h each. 12 fertilized eggs are deposited in the brood sac of 7–8-day-old mated females. To obtain crystals, embryos were gently extruded from the brood sac of a 54-day-old female. The midgut was isolated from each embryo by cutting off the head and the end of the abdomen, allowing the midgut to be extruded into insect Ringer’s solution. Supplementary Movie S1 shows how a cut made in the midgut allows its contents to be extruded by the contraction of muscles in the midgut wall. Crystals were collected in a Pasteur pipette and transferred to fresh sterile water, in which they are insoluble. Prior to X-ray diffraction experiments, crystals were cryoprotected in 20% glycerol and flash-cooled in liquid N_2_.

### 2.2. Crystallographic data-collection procedure for high-resolution crystals   

Data to 1.20 Å resolution were measured using a MAR CCD detector on beamline PXII at the Swiss Light Source (SLS), Villigen, Switzerland at a wavelength of 0.8349 Å (Pohl *et al.*, 2006 ▸). The sample-to-detector distance was set to 100 mm. All data collections were performed at cryo-temperatures using a 70 K nitrogen stream. Individual data sets were reduced with the d*TREK software (Pflugrath, 1999 ▸).

### 2.3. Recrystallization and data collection of solubilized protein   

Lili-Mip crystals obtained *in vivo* were solubilized in 50 m*M* sodium acetate pH 5.0. Size-exclusion chromatography was carried out on the solubilized protein using a Superdex 200 prep-grade column. The protein eluted as a homogenous and monodisperse fraction at 95.5 ml and was used for crystallization. Based on the Bio-Rad Gel Filtration Standard (Bio-Rad catalogue No. 151-1901), the Lili-Mip protein was calculated to elute as a monomer with a molecular weight of about 24 kDa. Purified Lili-Mip was crystallized in 25% PEG 10 000 at a concentration of 2 mg ml^−1^ and a temperature of 293 K. The high PEG concentration in the crystallization condition served as the cryoprotectant and hence additional PEG or glycerol were not added. The sizes of the recrystallized and the *in vivo* grown crystals were similar. The size of the crystal used for data collection was about 15 × 20 µm. X-ray diffraction data for these crystals was collected on the PROXIMA-1 beamline at the SOLEIL synchrotron, France, at a wavelength of 0.97857 Å. The sample-to-detector distance was set to 270.6 mm. All data collections were performed at cryotemperature using a 100 K nitrogen stream.

### 2.4. Structure determination by S-SAD   

*Ab initio* structure determination was performed by measuring the anomalous scattering signal of S atoms at a wavelength of 2.7 Å (4.6 keV). Data from seven crystals were merged to further enhance the anomalous signal. Reflections were collected with a Dectris PILATUS 2M-F detector on BL-1A at Photon Factory (PF), Tsukuba, Japan. The sample-to-detector distance was set to 60 mm. 720° of data were collected from each crystal, with an exposure time of 0.2 s per image and an oscillation angle of 0.2°. The sulfur substructure was determined using the *SHELXC*, *SHELXD* and *SHELXE* pipeline (Sheldrick, 1990 ▸). Three sites of anomalous scatterers, corresponding to two disulfide bridges and one Met S atom, were initially obtained. The correct hand was selected using the map correlation coefficient as the indicator. The initial calculated density from the correct hand was further refined with solvent histogram modifications using *SHELXE* and *Phaser* (McCoy *et al.*, 2007 ▸) from the *CCP*4 suite (Winn *et al.*, 2011 ▸). The model was built from this density through several cycles of secondary-structure fitting and side-chain assignment within *AutoSol* from the *PHENIX* suite (Adams *et al.*, 2010 ▸).

### 2.5. Structure refinements   

The three structures of Lili-Mip at 1.2, 1.75 and 2.5 Å resolution were refined through iterative cycles of restrained refinement using the *PHENIX* suite (Adams *et al.*, 2010 ▸) coupled with manual model building of electron densities generated with *Coot* (Emsley & Cowtan, 2004 ▸) until convergence (Table 1 ▸). Atomic coordinates and structure factors for the reported crystal structures have been deposited in the Protein Data Bank with accession codes 4nyr (Lili-Mip from S-SAD phasing), 4nyq (Lili-Mip at 1.2 Å resolution) and 5epq (*in vitro*-crystallized Lili-Mip). The difference maps showing the electron densities for the heterogeneous residues were prepared using *BUSTER* v.2.10.2 (Smart *et al.*, 2012 ▸). Alternate conformations of these heterogeneous residues were modelled with multiple occupancies using *Coot*. Fitting the sites with the heterogeneous residues from both Lili-Mip sequences completely refined the structure without additional density.

### 2.6. Mass spectrometry   

All protein samples were dissolved in 50% acetonitrile/0.3% trifluoroacetic acid for MALDI-TOF mass analysis. Mass analyses of the native protein were carried out with a Voyager-DE STR matrix-assisted laser desorption/ionization time-of-flight mass spectrometer (Perspective Biosystems, Framingham, Massachusetts, USA). The polycrystalline-layer method described by Beavis & Chait (1996 ▸) was used to apply the sample onto a gold-plated target with α-cyano-4-hydroxycinnamic acid as a matrix. Native and digested RNase B samples served as external calibration standards for the crystalline milk protein. The protein was deglycosylated using 10 units of 1 unit µl^−1^ N-glycosidase F (Roche) in 50 m*M* ammonium bicarbonate buffer pH 7.5 at 37°C for 20 h.

Glycosylated Lili-Mip samples were digested with trypsin or Asp-N and the deglycosylated sample with Asp-N alone using an enzyme:substrate ratio of 1:30. Samples containing 0.5 µg of the digested peptides were loaded onto a Zorbax 300SB-C18, 5 µm, 5 × 0.3 mm column using solvent *A* (100% acetonitrile with 0.1% formic acid) at a flow rate of 30 µl min^−1^ for 5 min. Following trapping and desalting, the peptides were transferred to an analytical column (Eksigent HALO C18 2.7 um, 90 Å, 100 × 0.5 mm) with a flow rate of 15 µl per min and resolved with a 40 min LC-MS run for Asp-N digested deglycosylated peptides and a 30 min LC-MS run for glycosylated peptides digested with trypsin or Asp-N. LC was carried out using a Eksigent nanoLC 425 and MS was performed using a AB Sciex TripleTOF 5600^+^. The raw data were acquired using the *Analyst* software v.1.6. The MS analysis was performed in IDA mode with ten MSMS experiments. Data processing was performed with the *ProteinPilot* software. The peptides obtained after the cleavage of the deglycosylated protein with Asp-N showed 100% sequence coverage for Lili-Mip 1 and Lili-Mip 2, and 53.5% for the Lili-Mip 3 sequence. The peptides obtained after trypsin cleavage of the glycosylated protein showed 71.6, 47.4 and 25.2% sequence coverage for Lili-Mip 1, Lili-Mip 2 and Lili-Mip 3, respectively. The peptides obtained after cleavage of the glycosylated protein with Asp-N showed 40.7 and 34.2% sequence coverage for Lili-Mip 1 and Lili-Mip 2, respectively.

### 2.7. Molecular dynamics simulations   

Deglycosylated native/ligand-unbound, oleic acid-bound and linoleic acid-bound Lili-Mip structures were generated *in silico*. MD simulations were performed using *GROMACS* v.4.6.4 (Hess *et al.*, 2008 ▸) with the GROMOS 43A1 force field (Schmid *et al.*, 2012 ▸; van Gunsteren *et al.*, 1996 ▸) for 30 ns. A cubic box was generated with a minimum distance of 10 Å between the protein and the edge of the box. The protein models were solvated with the SPC/E rigid water model (Berendsen *et al.*, 1987 ▸) and neutralized with sodium/chloride ions, depending on the net charge of the protein. Energy minimization was carried out with the steepest-descent algorithm until it converged with an *F*_max_ of no greater than 1000 kJ mol^−1^ nm^−1^. Position-restrained dynamics were performed for 2.5 ps. All bonds were constrained using the *Linear Constraint Solver* (*LINCS*) algorithm. The topologies of oleic acid and linoleic acid were generated using the *PRODRG* server (Schüttelkopf & van Aalten, 2004 ▸). The system was simulated under periodic boundary conditions with cutoffs of 10 Å for van der Waals terms. Long-range interactions were calculated using the particle mesh Ewald (PME) method. Principal component analysis (PCA) was also carried out using tools within the *GROMACS* package. The porcupine plots were generated using the mode-vectors script of *PyMOL* (DeLano, 2002 ▸).

### 2.8. Calculations of energetic values   

The energetic potential of Lili-Mip crystals was deduced by applying simple calculations of protein/sugar/lipid weight (Supplementary Table S4). To compare the energetic potential of Lili-Mip crystals with mammalian milks, the weight values were normalized to 100 g. The energetic values for the different milks are shown in Supplementary Table S3 (North Wales Buffalo, 2009 ▸). The content values of cholesterol and calcium were omitted for consistency.

## 3. Results   

Crystals extracted from the midgut of *D. punctata* embryos (Fig. 1 ▸) were dissolved and subjected to denaturing sodium dodecyl sulfate polyacrylamide gel electrophoresis (SDS–PAGE). The resulting gel revealed a streak, suggesting that there is significant heterogeneity (Supplementary Fig. S1*a*). Separation of the sample by free-flow electrophoresis (FFE) at constant pH resolved multiple peaks. Matrix-assisted laser desorption/ionization time-of-flight mass spectrometry (MALDI-TOF MS) spectra of both FFE-separated and dissolved crystals (Supplementary Fig. S2*a*) demonstrated significant heterogeneity of the samples. We postulate that this is owing to heterogeneous glycosylation. The mass spectra of the crystals following mannosidase treatment revealed that the glycosylation was mannose-enriched (Supplementary Fig. S2*b*). The lowest molecular weight was approximately 18.8 kDa, while the highest molecular weight was about 21.2 kDa, suggesting that glycosylation contributes 10–12% of the mass.

### 3.1. Crystal heterogeneity   

The midgut of a single cockroach embryo contains a large quantity of Lili-Mip crystals (Supplementary Movie S1). Significant heterogeneity among crystalline Lili-Mip was anticipated owing to multiple primary amino-acid sequences (Williford *et al.*, 2004 ▸), potential branched glycosylation and variable fatty-acid content (Ingram *et al.*, 1977 ▸). MALDI-TOF mass-spectrometric analysis of solubilized Lili-Mip from purified crystals confirmed the diverse molecular composition, as illustrated by a range of molecular weights from 22 to 30 kDa. The mass shifts might reveal variations among molecules within a single crystal as well as between different crystals. Liquid-chromatography quadrupole time-of-flight (LC-QTOF) mass-spectrometric analysis of the peptides generated by trypsin or Asp-N digestion of the solubilized glycosylated and deglycosylated Lili-Mip confirmed the presence of more than three different polypeptide sequences in the crystals. The sequences of three polypeptides (denoted Lili-Mip 1, 2 and 3), which share 80–90% identity, could be identified. An alignment highlighting the sequence similarity of Lili-Mip 1, 2 and 3 is shown in Supplementary Fig. S1(*b*). Furthermore, mass-spectrometric data indicated more than one variant sequence for some of the peptides (Table 2 ▸). The sequence with the largest coverage from mass-spectrometric analysis is called Lili-Mip 1. The complete sequence of Lili-Mip 1 can be considered as the major consensus sequence present, along with other variant peptides in the crystal, based on the fit to electron-density maps (see below).

Glycan analysis of solubilized Lili-Mip by mass spectrometry confirmed the presence of four N-linked glycosylation sites, Asn35, Asn66, Asn79 and Asn145, with Lili-Mip 3 containing only three sites (residue 145 is Lys in Lili-Mip 3). The core glycan structure is made up of two *N*-acetyl­glucosamine (NAG) molecules and one mannose (MAN) molecule. The presence of paucimannose and mannose-enriched glycan structures with variable branching were confirmed for Lili-Mip protein. Mass analysis further suggests that the bound ligand in Lili-Mip could be linoleic acid or oleic acid. The extent and degree of heterogeneity in the Lili-Mip protein is revealed from these spectrometric analyses.

### 3.2. X-ray structure determination   

The first Lili-Mip X-ray crystal structure was solved at 2.5 Å resolution by the single-wavelength anomalous dispersion (SAD) method using the anomalous scattering from S atoms in cysteines and methionines (Dauter *et al.*, 1999 ▸) of the protein. The sulfur SAD (S-SAD) structure was determined using data collected from multiple extracted crystals (Supplementary Movie S1). A complete native data set was collected from a single crystal that diffracted to 1.2 Å resolution. Additionally, several crystals were solubilized and recrystallized *in vitro*. A third data set was collected from an *in vitro*-grown Lili-Mip crystal. The starting model for refinement of these two data sets was the refined structure obtained from the S-SAD data. Crystallographic details are presented in Table 1 ▸. In all cases, Lili-Mip crystallized in the triclinic *P*1 lattice, with one molecule per asymmetric unit and unit-cell parameters *a* = 32.28, *b* = 33.22, *c* = 40.18 Å, α = 99.5, β = 100.28, γ = 104.11°.

The lack of redundancy from crystallographic symmetries makes phasing difficult for S-SAD structures of proteins that crystallize in the triclinic space group. To the best of our knowledge, structure determination from *P*1 crystals by this method has not been reported previously. The Lili-Mip protein sequence of 154 amino-acid residues contains four cysteines and one methionine. In order to obtain the highest anomalous signal and Bijvoet intensity ratio for reliable phasing, data were collected at a wavelength of 2.7 Å, corresponding to an X-ray energy of 4.59 keV. At this energy, the anomalous signal from S atoms corresponds to Δ*f*′′ = 1.51. The expected Bijvoet ratio for four free cysteines and one methionine at 4.59 keV was calculated to be 1.62%, which is higher than the reported Wang limit of 0.6% (Wang, 1985 ▸). High-redundancy data were collected from 11 isomorphous crystals. The phasing power of each independent data set was too weak for successful phase determination. As a consequence, various combinations of these data sets were initiated and compared in terms of phase determination and anomalous correlation (cutoff set at 30%). Seven data sets were retained, with an average redundancy, *R*_sym_ and mean *I*/σ(*I*) of 23.0, 0.067 and 38.1, respectively (Table 1 ▸). From these combined data, structure determination and automatic density modification resulted in maps that could be used to build the initial structure. The final refined structure has a total of 139 out of 151 residues built, with *R*_work_ and *R*_free_ values of 15.5 and 23.3%, respectively. The large difference is most likely owing to the merging of seven different data sets for phasing that come together with slight non-isomorphism. This is also reflected in the difference in unit-cell parameters of the independently processed data from the individual crystals. While there is very little difference (±0.1 Å) in the *a* and *c* dimensions, there is a difference of ±0.3 Å in the *b* direction. Similarly, the largest variation in the angle is in the α angle (±0.3°). This combined data set was also used for the refinement of the structure. The limitation of the data to 2.5 Å resolution is, however, not owing to this non-isomorphism. Practical experimental considerations for data collection at 2.7 Å wavelength and a crystal-to-detector distance of 60 mm restricted data collection to 2.5 Å resolution.

### 3.3. Structure of Lili-Mip   

**Figure 2:**
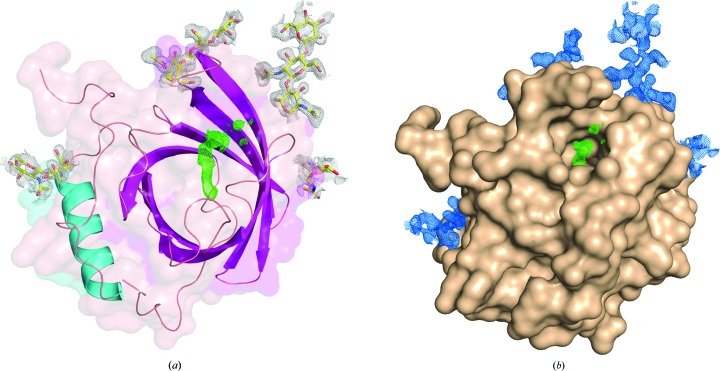
Crystal structure of Lili-Mip. (*a*) Cartoon diagram of the Lili-Mip structure consisting of one C-terminal α-helix (light blue) and nine β-strands (magenta) that form a barrel to loosely coordinate the lipid. The N-glycans (yellow) at the four glycosylation sites are modelled in 2*F*_o_ − *F*_c_ electron density (white). (*b*) Surface view of Lili-Mip showing the 2*F*_o_ − *F*_c_ electron-density map (blue) contoured at 1× r.m.s. for the N-glycans at Asn35, Asn79 and Asn145, and 0.5σ for that at Asn66. The wire mesh (green) in the middle of the structures in both panels is the difference map showing density for the lipid drawn at 3.0σ.

A structure of Lili-Mip from the cockroach midgut crystals reveals a lipocalin fold (Fig. 2 ▸ *a*). Members of the lipocalin family typically accommodate lipophilic ligands in a cavity shaped by a common fold composed of a central β-barrel comprising eight antiparallel strands with four structurally variable peptide loops at the aperture entrance (Salier *et al.*, 2004 ▸; Skerra, 2000 ▸). The 2*F*_o_ − *F*_c_ and *F*_o_ − *F*_c_ electron-density maps of the 1.2 Å resolution structure revealed densities for glycosylation at Asn35, Asn66, Asn79 and Asn145. At positions 35 and 79, one β-mannose (BMA) and two NAGs were identified. At Asn145 two NAG molecules could be modelled in the density, and one NAG was modelled at Asn66 (Fig. 2 ▸ *b*). The NAG at Asn66 and the two BMAs at Asn35 and Asn79 are partially disordered. The NAG molecules are linked to one another and to the mannose molecules *via* β(1→4) glycosidic bond linkages. The crystallographic data concurred with the MS data for the presence of four N-linked glycosylation sites.

**Figure 3:**
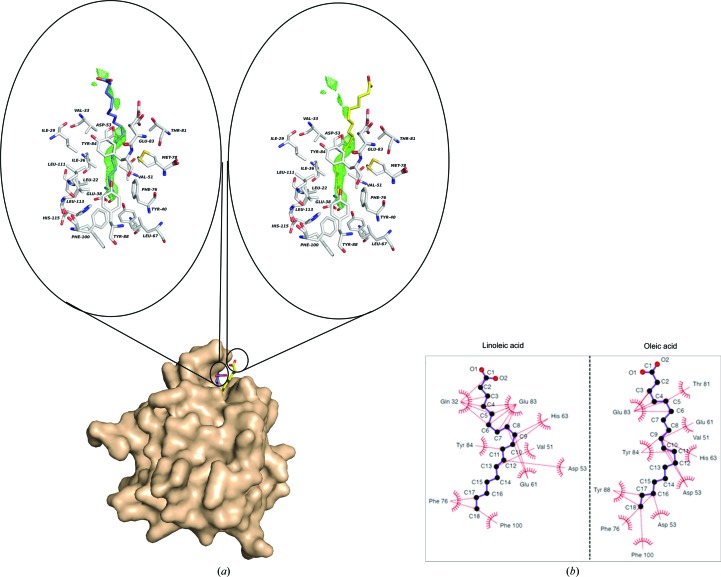
Lipid binding to Lili-Mip. (*a*) Close-up view of the interface between the lipids (linoleic acid, purple; oleic acid, yellow) and the hydrophobic cavity. Residues involved in the formation of the cavity are modelled and labelled. The *F*_o_ − *F*_c_ electron-density map (drawn at 3.0σ) for the lipids in the binding cavity is shown in green. As mentioned in the text, the last few C atoms and the charged group are disordered and different in the different structures. The electron-density map depicted is using data from PDB entry 4nyq. (*b*) Two-dimensional projection of lipid coordination by Lili-Mip residues: *LIGPLOT* diagram (Wallace *et al.*, 1995 ▸). Atoms of the lipid are labelled in black and Lili-Mip residues are shown in red. The direction of the hydrophobic interactions between each atom of the lipid and Lili-Mip is represented.

All higher resolution models possibly coordinate different lipids within a hydrophobic pocket, namely linoleic acid or oleic acid (Fig. 3 ▸ *a*). The cavity in the Lili-Mip structure is 15 Å deep, with a volume of 727 Å^3^, and can accommodate up to 18-carbon fatty-acid chain ligands. Upon binding free lipids (Fig. 3 ▸ *b*), approximately 832 Å^2^ of solvent-accessible surface area is buried in Lili-Mip, and only the polar head group and possibly an adjacent C atom sit outside the binding pocket. In the crystal structures, the head group of the lipid and several C atoms closest to the head group are disordered. The approximate average distance between the residues forming the Lili-Mip hydrophobic cavity and linoleic acid or oleic acid is summarized in Supplementary Table S2. 12 residues (Val33, Ile36, Asp53, Glu61, His63, Phe76, Met78, Thr81, Glu83, Tyr84, Tyr88 and Phe100) form a foundation for the lipid pronged interaction with Lili-Mip. Four aromatic residues (Phe76, Tyr84, Tyr88 and Phe100), combined with Leu113 and Glu38, delimit the deepest depression, notably through the formation of a stable π-stacking of Tyr88 and Phe100 rings that restrict the length of the lipid.

### 3.4. Heterogeneity in the Lili-Mip amino-acid sequence   

**Figure 4:**
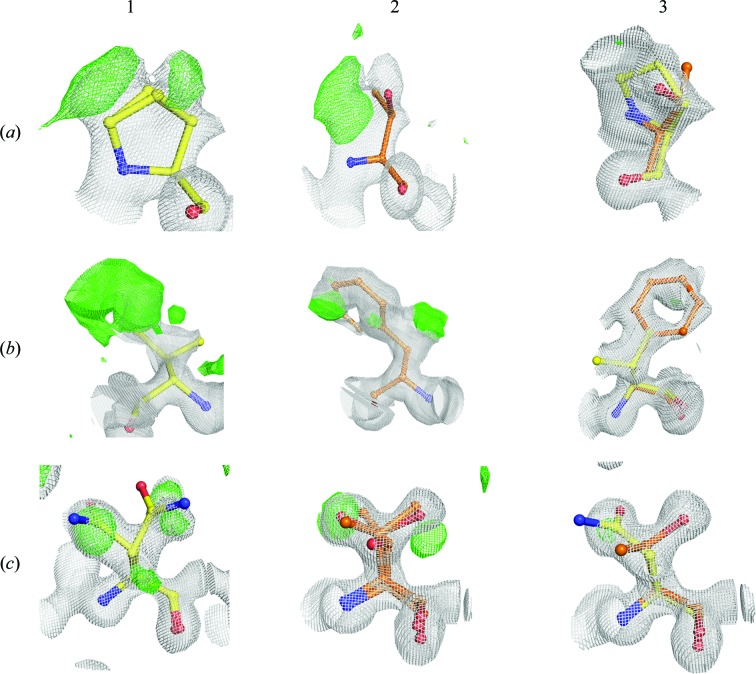
2*F*_o_ − *F*_c_ (white) and *F*_o_ − *F*_c_ (green) electron-density maps for three residues where heterogeneity is observed by crystallography and mass spectrometry. All 2*F*_o_ − *F*_c_ maps are contoured at 1 × r.m.s. values. (*a*) Residue 12 is Pro in Lili-Mip 1 and Thr in Lili-Mip 2. The difference map (green) is at the 3σ level. (*b*) Residue 39 is Val in Lili-Mip 1 and Phe in Lili-Mip 2. The difference map (green) is at the 2σ level in the first panel to show the complete ring of Phe. (*c*) Residue 50 is Asn in Lili-Mip 1 and Thr in Lili-Mip 2. The difference map (green) is contoured at the 3σ level. In all three figures, panel 1 shows the additional densities after refining only the Lili-Mip 1 sequence (residues in yellow) and panel 2 after refining only the Lili-Mip 2 sequence (residues in orange). Panel 3 shows that after refining with both Lili-Mip 1 and 2 sequences, no additional densities are observed. The electron-density map depicted is using data from PDB entry 4nyq.

As refinement progressed, small ambiguities in the electron densities of several side chains (Fig. 4 ▸) suggested that the crystals contained multiple proteins with differing primary amino-acid sequences, consistent with previous characterizations of the milk proteins (Williford *et al.*, 2004 ▸). Considering the high *B* factors and disorder among amino-acid side chains, heterogeneity for six of 28 residues could be visualized clearly in the 2*F*_o_ − *F*_c_ and *F*_o_ − *F*_c_ electron-density maps. At each position, after modelling and refining the residue from one of the sequences with partial occupancy, additional density was observed for the corresponding residue from another sequence. Fig. 4 ▸ shows the electron-density maps for residues 12, 39 and 50. Despite several attempts, the difference density could not be accounted for by modelling alternate conformations for these residues. Visualization of the OMIT maps obtained after deletion of these residues revealed features corresponding to the presence of multiple sequences. It is rare that crystals of heterogeneous proteins diffract to atomic resolution, owing to their intrinsic disorder. Amino-acid sequence heterogeneity was observed in the structures determined from all three data sets. Two of the data sets were completely collected from a single crystal, while one data set (used for S-SAD) was collected from multiple crystals. We therefore exclude the possibility that this heterogeneity results solely owing to the merging of data sets from multiple crystals for the S-SAD structure determination. We can conclude from these observations that the monomer obtained in each asymmetric unit is a space average from all of the sequences, *i.e.* the crystal is made from packing of proteins with multiple sequences.

### 3.5. Crystal packing   

**Figure 5:**
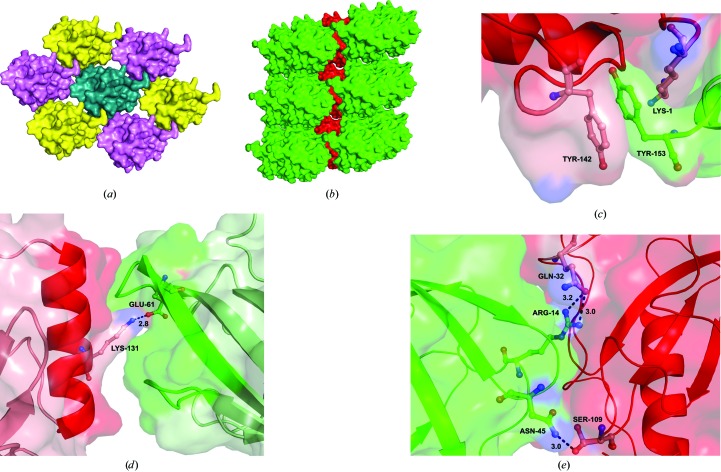
Crystal packing in Lili-Mip. (*a*) The arrangement of molecules in a plane. Each molecule is surrounded by six other molecules. (*b*) Overall crystal packing showing a sheath within a cylinder arrangement. (*c*) The π–π stacking interaction between Tyr153 and Tyr142 of two neighbouring molecules. (*d*) The C-­terminal helix interaction with a groove formed by the loops and β-strand in the opening of the ligand-binding site through a salt bridge between Lys131 and Glu61. (*e*) The third interacting region between neighbouring molecules through hydrogen bonds between Asn45 and Ser109 as well as Arg14 and Gln32.

Each molecule of Lili-Mip is surrounded by six molecules in one plane (Fig. 5 ▸ *a*) and is sandwiched between two other molecules: one above and one below. This gives the appearance of sheaths of molecules enclosed within a cylinder formed by the six molecules in one plane (Fig. 5 ▸ *b*). There are three regions on the surface of one molecule that interact tightly with the neighbouring molecules. The first set of interactions consists of a π–π stacking interaction between the C-terminal Tyr153 of one molecule and Tyr142 of the neighbouring molecule. Interestingly, Tyr153 of the first molecule and Lys1 of the second molecule are proximal to one another without making any apparent inter­actions (Fig. 5 ▸ *c*). In the second region, the C-terminal helix (residues 123–136) of one molecule binds to a groove formed by a loop (residues 78–84), a *β*-strand (residues 59–65) and another loop (residues 55–58) in the neighbouring molecule. Lys131, present in the C-terminal helix, forms a salt-bridge interaction with Glu61, which is buried in the groove (Fig. 5 ▸ *d*). The third interaction area is larger than the other two regions. Here, Asn45 from one molecule forms a hydrogen bond with Ser109 in the neighbouring molecule. Similarly, Arg14 of the first molecule forms two hydrogen bonds to Gln32 in the neighbouring molecule (Fig. 5 ▸ *e*). Together, these three interactions create a compact crystallographic lattice of well–ordered molecules. Interestingly, the heterogeneous residues are mostly located on the surface but are not involved in crystal packing.

### 3.6. Molecular dynamics simulation studies   

**Figure 6:**
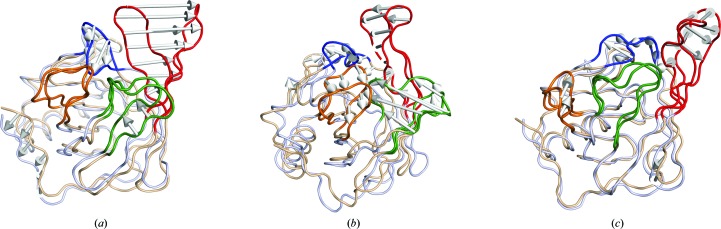
Porcupine plots showing relative motions of the four regions in the deglycosylated models of (*a*) native, (*b*) oleic acid-bound and (*c*) linoleic acid-bound Lili-Mip. Regions I, II, III and IV are coloured blue, red, green and orange, respectively.

Molecular breathing is a phenomenon where the gorge formed by the β-strands remains open to the solvent and ready to accept lipids (Supplementary Fig. S3*a*) while there are specific loops that open and close at the entrance. In the bound crystal structures, the mouth of the gorge shows an opening diameter of ∼10 Å, which suggests that mainly linear lipids would fit inside the cavity without major remodelling of the protein conformation. To understand the mechanism of lipid entrance and exit from the gorge, molecular dynamics simulations were carried out using the *in silico*-generated deglycosylated proteins. 30 ns simulations were performed using three different Lili-Mip starting structures: native/ligand-unbound (DglyNat), oleic acid-bound (DglyOla) and linoleic acid-bound (DglyEic). A comparison of the root-mean-square deviation (r.m.s.d.) of the backbones during the three 30 ns simulations (Supplementary Fig. S3*b*) shows that the DglyNat, DglyOla and DglyEic systems stabilize after about 15, 9 and 3 ns, respectively. A comparison of the root-mean-square fluctuation (r.m.s.f.) values for the Cα atoms of Lili-Mip in the three simulations is shown in Supplementary Fig. S3(*c*). There are four regions [designated I (residues 30–35), II (residues 50–65), III (residues 75–85) and IV (residues 102–112) in Supplementary Fig. S3*c*] with higher r.m.s.f. values when compared with other regions of the structure. Interestingly, these four regions surround the opening of the lipid-binding pocket (Supplementary Fig. S3*a*). To understand the relative opening and closing motions of these four regions during the three simulations, principal component analysis (PCA; Amadei *et al.*, 1993 ▸) was carried out and porcupine plots of the eigenvectors were generated from the simulations (Fig. 6 ▸). In a porcupine plot, two extreme conformations of a protein are represented that show the maximum extent of movement in different regions during a simulation. As highlighted in Fig. 6 ▸, the four regions surrounding the ligand-binding pocket show maximum displacement/fluctuations in DglyNat compared with DglyOla or DglyEic. Such inherent higher fluctuations in the ligand-unbound structure suggest a plausible mechanism of molecular breathing in Lili-Mip where the loops open and close until a lipid binds. Binding of the lipid stabilizes the closed form.

## 4. Discussion   

*D. punctata* provides one of the few examples of viviparity among insects. Similar to mammals, the mother supplies nutrition in the form of a milk secretion, known as Lili-Mip, to the 9–12 developing embryos in her brood sac. Lili-Mip serves as a complete nutrient by providing all of the essential amino acids, carbohydrates from the attached glycans, and lipids through chaperoning linoleic and oleic acids. Lili-Mips are the major nutrient source for developing embryos prior to birth. After ingestion by the embryos, its increasing concentration in the midgut facilitates the crystallization of Lili-Mip. Significant heterogeneity was observed in the primary protein structure, glycosylation and lipid content of Lili-Mip; however, the precise role of heterogeneity optimized for crystallization in a single lattice is currently unclear.

This is the first report of direct crystallographic phasing and structure determination from a crystal naturally grown *in vivo* rather than *in vitro* from overexpressed proteins. The atomic resolution structure of crambin is an example of the crystallization and structure determination of a protein with micro-heterogeneity in its primary sequence (Hendrickson & Teeter, 1981 ▸; Teeter & Roe, 1993 ▸). While there are examples of minor heterogeneity in crystal structures in protein sequence, glycosylation and ligand binding independently, to the best of our knowledge, there is no reported crystal structure to date that has heterogeneity in protein sequence, carbohydrate and lipid content together. Certainly not surprisingly, most structures with heterogeneity do not diffract to high resolution. We present here a unique example of crystals with significant heterogeneity that diffract to atomic resolution. The extent of glycosylation associated with Lili-Mip crystals is remarkable, as *in vitro* crystallization of glycosylated proteins is known to be problematic.

Upon structure determination, Lili-Mip was found to belong to the family of lipocalin-like proteins. Superimposition of Lili-Mip structures with different models of lipocalins resulted in r.m.s.d. values ranging from 3.90 to 15.38 Å, confirming the highly redundant nature of this fold in the form of a calix (Supplementary Table S1). The major differences among these proteins reside in the conformation of the hydrophobic cavity used for lipid coordination, which determines the type of ligand that can be accommodated, such as specific sets of lipids, steroids, bilins or retinoids (Flower *et al.*, 1993 ▸). The structures suggest that the energetics of crystallization (albeit crystallization of a heterogeneous mixture) create a storage and release mechanism that is simply concentration-dependent in order to supply nutrients as they are needed.

Compositional analysis of the milk secreted by pregnant *D. punctata* females indicated that lipids contribute 16–22% of the dry weight, with cholesterol being the only steroid and linoleic acid being the most abundant fatty acid (Ingram *et al.*, 1977 ▸). Linoleic acid is essential to the diet of most insects, and in other animals lipocalins are known to transport hydrophobic molecules, such as cholesterol and linoleic acid, that cannot be synthesized by insects *de novo* (Dadd, 1973 ▸; Salier *et al.*, 2004 ▸; Skerra, 2000 ▸; Flower *et al.*, 1993 ▸). Mass analysis of Lili-Mip crystals shows the presence of linoleic and oleic acids. In the crystal structure, we observed a long fatty-acid chain in the barrel that we surmise to be either linoleic acid or oleic acid (Fig. 3 ▸). Molecular dynamics simulation studies suggested that linoleic acid-bound Lili-Mip had less fluctuation than oleic acid-bound or native Lili-Mip. Furthermore, the binding of oleic *versus* linoleic acid results in conformational changes among the buried residues in the core of the lipocalin fold. Residues Val51, Val65, Thr81, Ser86, Phe100 and Leu113 line the binding pocket and show multiple conformations, suggesting that both oleic acid-bound and linoleic acid-bound proteins co-exist in the crystal. Also, all heterogeneous residues of the protein mixture are located on the surface of and not inside the barrel, suggesting a conserved binding pocket.

The high growth rate of *D. punctata* larvae from birth to reproductive maturity in 43–52 d (Willis *et al.*, 1958 ▸; Stay & Coop, 1973 ▸), compared with 160 d for the ovoviviparous *Rhyparobia maderae* larvae, might be a consequence of the exceptional energetic potential of the Lili-Mip crystals. Between the start of yolk formation in the ovary and the birth of *D. punctata* embryos, the protein content increases 600-fold (Stay & Coop, 1973 ▸); this is approximately nine times more than the protein in *R. maderae* larvae (Dejmal & Brookes, 1968 ▸). The high protein content is attributable to the Lili-Mip provided by the brood sac and its storage as crystals in the embryo midgut. A single midgut crystal of Lili-Mip accounts for approximately 3.7 × 10^−5^ J and corresponds to more than three times the energy provided by the equivalent masses of mammalian milks from several species (Supplementary Table S3). The formation of casein micelles is an important functional feature for maintaining mammalian milk with high protein content yet low viscosity (Slattery & Evard, 1973 ▸). In contrast, the high protein content of *D. punctata* milk is achieved by crystallization of Lili-Mip in the embryo midgut.

There are numerous examples in the literature where *in vivo* protein crystallization is regulated by mechanisms such as ionic changes, proteolysis and chaperone proteins (Doye & Poon, 2006 ▸). Crystallization is induced by increasing the protein concentration to the levels of supersaturation that leads to nucleation, followed by an orderly assembly. The *in vitro* recrystallization of the solubilized Lili-Mip crystals, using only higher molecular-weight polyethylene glycol, demonstrated the high propensity of this protein for crystallization. The strong interactions observed between the related molecules in the lattice might be an indication of an efficient nucleation phenomenon. Analysis of the packing among the proteins in the crystal provides a possible explanation for the high crystallizability of this protein. Presumably, as the embryos begin to consume the food, the concentration of Lili-Mip in solution decreases, causing the crystals to dissolve. Equilibrium is maintained, allowing the release of food as there is a need for nutrients. In other words, storage of food in crystalline form not only allows a high concentration of food to be stored, but also provides a mechanism for the controlled release of nutrients as they are needed. Understanding the molecular structure of these *in vivo*-grown protein crystals allows us to appreciate how the principles of thermodynamics (crystal packing) and kinetics (equilibrium between crystalline and solution states) are exquisitely utilized in biology to provide an evolutionary advantage.

## 5. Related literature   

The following references are cited in the Supporting Information for this article: Robert & Gouet (2014 ▸) and Sievers *et al.* (2011 ▸).

## Supplementary Material

PDB reference: Lili-Mip, high-resolution structure, 4nyq

PDB reference: S-SAD structure, 4nyr

PDB reference: recrystallized structure, 5epq

Supplementary Movie S1. Extraction of in vivo-grown crystals. Crystals of Lili-Mip are extracted from the midgut of D. punctata embryos by dissection. Crystals pouring out of the midgut in large quantities are stored in sterile water.. DOI: 10.1107/S2052252516008903/jt5013sup2.mov

Supplementary Movie S2. Movements observed in regions I to IV during the 30 ns of MD simulation with native protein.. DOI: 10.1107/S2052252516008903/jt5013sup3.mov

Supplementary Movie S3. Movements observed in regions I to IV during the 30 ns of MD simulation with the oleic acid-bound structure.. DOI: 10.1107/S2052252516008903/jt5013sup4.mov

Supplementary Movie S4. Movements observed in regions I to IV during the 30 ns of MD simulation with the linoleic acid-bound structure.. DOI: 10.1107/S2052252516008903/jt5013sup5.mov

## Figures and Tables

**Table 1:** X-ray data-collection and refinement statistics

	High resolution (PDB entry 4nyq)	SAD phasing (PDB entry 4nyr)	Recrystallized (PDB entry 5epq)
Data collection			
Beamline	PXII, SLS	BL-1A, PF	PROXIMA-1, SOLEIL
Space group	*P*1	*P*1	*P*1
Unit-cell parameters			
*a* (Å)	32.3	32.3	32.2
*b* (Å)	33.2	33.2	33.3
*c* (Å)	40.1	40.2	39.9
α (°)	99.1	99.5	99.2
β (°)	100.2	100.3	100.0
γ (°)	103.7	104.1	103.8
Resolution (Å)	27–1.2 (1.23–1.20)	50–2.5 (2.54–2.50)	50–1.75 (1.86–1.75)
*R*_meas_	0.06 (0.10)	0.067 (0.08)	0.19 (0.53)
〈*I*/σ(*I*)〉	9.0 (2.4)	38.1 (22.4)	4.0 (1.7)
Completeness (%)	95.6 (93.9)	99.6 (96.7)	95.1 (90.8)
Multiplicity	3.15 (3.07)	23 (15.8)	1.8 (1.8)
Anomalous correlation (%)	—	44 (27)	—
Anomalous signal	—	1.12 (0.66)	—
Refinement			
Resolution (Å)	27.4–1.2 (1.23–1.20)	38.58–2.5 (2.56–2.49)	38.4–1.75 (1.86–1.75)
No. of reflections	46776	4876	15193
*R*_work_/*R*_free_	0.158/0.201	0.155/0.233	0.179/0.220
No. of atoms			
Protein	1481	1248	1439
Ligand/ion	54	76	28
Water	210	88	180
*B* factors (Å^2^)			
Protein	17.8	17.2	16.5
Ligand/ion	53.3	—	52.0
Water	37.4	16.8	31.4
R.m.s. deviations			
Bond lengths (Å)	0.005	0.013	0.007
Bond angles (°)	1.06	1.91	0.964
Ramachandran analysis (%)			
Most favoured	99.4	96.0	100.0
Allowed	0.6	4.0	0.0

**Table 2:** Lili-Mip sequence-variant peptides observed by mass-spectrometric analysis The underlined peptide sequences or single residues represent the variant sequences. Peptides generated by trypsin digestion are coded ‘T’. Peptides generated by Asp-N digestion are coded ‘A’.

Peptide	Lili-Mip 1	Lili-Mip 2	Lili-Mip 3	Sequence variants
T1-2	KEPCPPENLQLTPR	KEPCPPENLQLTPR	KEPCPPENLQLPPR	
A3	DITEFYSAHGN	DITEFYSAHGN	DITEVYSAHGN	DITEFYSAHDN
				DITEVYDARGN
				DITEVYNARGN
				DITEVYTTRGN
A4	DYYGTVT	DYYGTVT	DYYGNVT	
A5	DYSPEYGLEAHRV	DYSPEYGLEAHRV		DYSPEYGLQTHRV
				DYSPEFGLQTHRV
				DYSPEYGLEEHRVV
				DYSPEYGLEAHQ
A9-10	DSKYEILAVDK	DSEYEILAVDK		DSWYEILAVDK
				DTDYQILAVDK
A12/T12-13-14	DVIKRVKKALKNVCL/DVIKRVKK	DIIKRVKKSLKNVCL/DIIKRVKK		DIIKSVK
A13	DYKYFGD		DYKYFSK	DDTSVHCHYVE
A14-15	DDTSVPCHYVE		DDTSVHCRYLE	DDTSVPCN
				DDTSVPCH†
				DDTSVPCHY†
				DDTSVHCH†
				DDTSVHCHY†
				DDTSVHCHYV†

† C-terminal amino-acid loss variant.
